# Supplementary material for: The Systems Biology Research Tool: evolvable open-source software
Source: BMC Syst Biol. 2008 Jun 29;2:55. doi: 10.1186/1752-0509-2-55 (PMC2446383; doi:10.1186/1752-0509-2-55)
Supplement: Additional file 1 — SBRT Archive. An archive of the current version of the Systems Biology Research Tool. [file 1752-0509-2-55-S1.zip › sbrt-1.4.0/doc/developers_guide/api/sbrt/kernel/math/optimization/class-use/OptimizationSense.html]

Uses of Class sbrt.kernel.math.optimization.OptimizationSense


|  |  |  |
| --- | --- | --- |
| **Overview** \| **Package** \| **Class** \| **Use** \| **Tree** \| **Deprecated** \| **Index** \| **Help** | |  |
| PREV   NEXT | **FRAMES**    **NO FRAMES**     **All Classes** |  |


---


## **Uses of Class sbrt.kernel.math.optimization.OptimizationSense**

| Packages that use OptimizationSense | |
| --- | --- |
| **sbrt.ext.cplex** | Provides a class that allows communication with CPLEX. |
| **sbrt.ext.glpk** | Provides a class that allows communication with GLPK. |
| **sbrt.kernel.fba.comp** | Provides classes and interfaces for performing Flux Balance Analysis computations. |
| **sbrt.kernel.fba.proc** | Provides classes and interfaces for executing Flux Balance Analysis processes. |
| **sbrt.kernel.math.optimization** | Provides classes and interfaces for creating objects relevant to solving optimization problems. |
| **sbrt.shell.mng.fba** | Provides classes and interfaces for creating Flux Balance Analysis process managers. |
| **sbrt.shell.text.math** | Provides classes and interfaces for defining formats for mathematical objects. |

Uses of OptimizationSense in sbrt.ext.cplex

| Methods in sbrt.ext.cplex with parameters of type OptimizationSense | |
| --- | --- |
| `void` | `CplexSolver.setObjectiveFunction(LinearComb<? extends C> objective, OptimizationSense sense)`             Sets the objective function and optimization sense to the provided values. |
| `void` | `CplexSolver.setObjectiveFunction(MathExpr<? extends C> objective, OptimizationSense sense)`             Sets the objective function and optimization sense to the provided values. |
| `void` | `CplexSolver.setObjectiveFunction(QuadraticExpr<? extends C> objective, OptimizationSense sense)`             Sets the objective function and optimization sense to the provided values. |
| `void` | `CplexSolver.setOptimizationSense(OptimizationSense sense)`             Sets the optimization sense. |

Uses of OptimizationSense in sbrt.ext.glpk

| Methods in sbrt.ext.glpk with parameters of type OptimizationSense | |
| --- | --- |
| `void` | `GlpkSolver.setObjectiveFunction(LinearComb<? extends C> expression, OptimizationSense sense)`             Sets the objective function and optimization sense to the provided values. |
| `void` | `GlpkSolver.setObjectiveFunction(MathExpr<? extends C> expression, OptimizationSense sense)`             Sets the objective function and optimization sense to the provided values. |
| `void` | `GlpkSolver.setOptimizationSense(OptimizationSense sense)`             Sets the optimization sense. |

Uses of OptimizationSense in sbrt.kernel.fba.comp

| Methods in sbrt.kernel.fba.comp that return OptimizationSense | |
| --- | --- |
| `OptimizationSense` | `RxnDeletionComputer.getOptimizationSense()`             Returns the current optimization sense. |
| `OptimizationSense` | `FbaOptException.getSense()`             Returns the sense, or direction, of the attempted optimization. |

| Methods in sbrt.kernel.fba.comp with parameters of type OptimizationSense | |
| --- | --- |
| `FbaOptComputer.Results` | `FbaOptComputer.getOptimal(MathExpr<?> objective, OptimizationSense sense)`             Returns the results of computing the optimal value of the provided objective function. |
| `FbaOptComputer.Results` | `FbaOptComputer.getOptimal(java.lang.String reactionName, OptimizationSense sense)`             Returns the results of computing the optimal flux of the reaction with the provided name. |
| `void` | `RxnDeletionComputer.setOptimizationSense(OptimizationSense sense)`             Sets the optimization sense to the provided value. |

Constructors in sbrt.kernel.fba.comp with parameters of type OptimizationSense

`FbaOptException(MathExpr<?> objectiveFunction,
OptimizationSense sense,
ProgramSolverStatus status)`
  
          Constructs a new FBA optimization exception.

`FbaOptException(MathExpr<?> objectiveFunction,
OptimizationSense sense,
ProgramSolverStatus status,
java.lang.String message)`
  
          Constructs a new FBA optimization exception.

Uses of OptimizationSense in sbrt.kernel.fba.proc

| Methods in sbrt.kernel.fba.proc that return OptimizationSense | |
| --- | --- |
| `OptimizationSense` | `CvFbaOptimizationExperiment.getOptimizationSense()`             Returns the current optimization sense. |
| `OptimizationSense` | `ObjectiveFunctionAnalysis.getOptimizationSense()`             Returns the current optimization sense. |
| `OptimizationSense` | `CatalystDeletionProcess.getOptimizationSense()`             Returns the current optimization sense. |
| `OptimizationSense` | `RxnDeletionProcess.getOptimizationSense()`             Returns the current optimization sense. |
| `OptimizationSense` | `CvRxnDeletionProcess.getOptimizationSense()`             Returns the current optimization sense. |
| `OptimizationSense` | `StoichVariationProcess.getOptimizationSense()`             Returns the current optimization sense. |
| `OptimizationSense` | `CvObjectiveFunctionProcess.getOptimizationSense()`             Returns the current optimization sense. |
| `OptimizationSense` | `FbaOptimizationProcess.getOptimizationSense()`             Returns the current optimization sense. |
| `OptimizationSense` | `CvCatalystDeletionProcess.getOptimizationSense()`             Returns the current optimization sense. |

| Methods in sbrt.kernel.fba.proc with parameters of type OptimizationSense | |
| --- | --- |
| `void` | `CvFbaOptimizationExperiment.setOptimizationSense(OptimizationSense sense)`             Sets the optimization sense to the provided value. |
| `void` | `ObjectiveFunctionAnalysis.setOptimizationSense(OptimizationSense sense)`             Sets the optimization sense to the provided value. |
| `void` | `CatalystDeletionProcess.setOptimizationSense(OptimizationSense sense)`             Sets the optimization sense to the provided value. |
| `void` | `RxnDeletionProcess.setOptimizationSense(OptimizationSense sense)`             Sets the optimization sense to the provided value. |
| `void` | `CvRxnDeletionProcess.setOptimizationSense(OptimizationSense sense)`             Sets the optimization sense to the provided value. |
| `void` | `StoichVariationProcess.setOptimizationSense(OptimizationSense sense)`             Sets the optimization sense to the provided value. |
| `void` | `CvObjectiveFunctionProcess.setOptimizationSense(OptimizationSense sense)`             Sets the optimization sense to the provided value. |
| `void` | `FbaOptimizationProcess.setOptimizationSense(OptimizationSense sense)`             Sets the optimization sense to the provided value. |
| `void` | `CvCatalystDeletionProcess.setOptimizationSense(OptimizationSense sense)`             Sets the optimization sense to the provided value. |

Uses of OptimizationSense in sbrt.kernel.math.optimization

| Methods in sbrt.kernel.math.optimization that return OptimizationSense | |
| --- | --- |
| `static OptimizationSense` | `OptimizationSense.valueOf(java.lang.String name)`             Returns the enum constant of this type with the specified name. |
| `static OptimizationSense[]` | `OptimizationSense.values()`             Returns an array containing the constants of this enum type, in the order they're declared. |

| Methods in sbrt.kernel.math.optimization with parameters of type OptimizationSense | |
| --- | --- |
| `void` | `LinearProgramSolver.setObjectiveFunction(LinearComb<? extends C> expression, OptimizationSense sense)`             Sets the objective function and optimization sense to the provided values. |
| `void` | `ProgramSolver.setObjectiveFunction(MathExpr<? extends C> expression, OptimizationSense sense)`             Sets the objective function and optimization sense to the provided values. |
| `void` | `QuadraticProgramSolver.setObjectiveFunction(QuadraticExpr<? extends C> expression, OptimizationSense sense)`             Sets the objective function and optimization sense to the provided values. |
| `void` | `ProgramSolver.setOptimizationSense(OptimizationSense sense)`             Sets the optimization sense. |

Uses of OptimizationSense in sbrt.shell.mng.fba

| Methods in sbrt.shell.mng.fba that return OptimizationSense | |
| --- | --- |
| `static OptimizationSense` | `FbaOptManager.getOptimizationSense(ManagerInput<java.lang.String,java.lang.String> input)`             Parses and returns the value for `FbaOptManager.OPTIMIZATION_SENSE` in the provided manager input. |

Uses of OptimizationSense in sbrt.shell.text.math

Methods in sbrt.shell.text.math with type parameters of type OptimizationSense

`<S extends OptimizationSense>
  
java.lang.String`

`SenseFormat.format(S obj)`
  
          Returns a formatted string representation of the
provided optimization sense.

| Methods in sbrt.shell.text.math that return OptimizationSense | |
| --- | --- |
| `OptimizationSense` | `SenseFormat.parse(java.lang.String source)`             Parses the provided string and returns its corresponding object. |

---


|  |  |  |
| --- | --- | --- |
| **Overview** \| **Package** \| **Class** \| **Use** \| **Tree** \| **Deprecated** \| **Index** \| **Help** | |  |
| PREV   NEXT | **FRAMES**    **NO FRAMES**     **All Classes** |  |


---
